# Synergistic Potential of Antibiotics with Cancer Treatments

**DOI:** 10.3390/cancers17010059

**Published:** 2024-12-28

**Authors:** Giuseppe Nardo, Pan Pantziarka, Matteo Conti

**Affiliations:** 1 Fondazione IRCCS Istituto Nazionale dei Tumori, Via Venezian 1, 20133 Milano, Italy; 2 Anticancer Fund, 1860 Meise, Belgium; pan.pantziarka@anticancerfund.org; 3 George Pantziarka TP53 Trust, London E1 8FA, UK; 4 Dipartimento Sanità Pubblica, AUSL Imola, Viale Amendola 8, 40026 Imola, Italy; matteo.conti@ausl.imola.bo.it

**Keywords:** antibiotics, microbiota, microbiome, immunotherapy, chemotherapy, radiotherapy

## Abstract

Intratumoral microbiota, the diverse community of microorganisms residing within tumor tissues, represent an emerging and intriguing field in cancer biology. These microbial populations are distinct from the well-studied gut microbiota, offering novel insights into tumor biology, cancer progression, and potential therapeutic interventions. Recent studies have explored the use of certain antibiotics to modulate intratumoral microbiota and enhance the efficacy of cancer therapies, showing promising results. Antibiotics can alter intratumoral microbiota’s composition, which may have a major role in promoting cancer progression and immune evasion. Certain bacteria within tumors can promote immunosuppression and resistance to therapies. By targeting these bacteria, antibiotics can help create a more favorable environment for chemotherapy, targeted therapy, and immunotherapy to act effectively. Some bacteria within the tumor microenvironment produce immunosuppressive molecules that inhibit the activity of immune cells. The combination of antibiotics and other cancer therapies holds significant promise for creating a synergistic effect and enhancing the immune response against cancer. In this review, we analyze several preclinical studies that have been conducted to demonstrate the synergy between antibiotics and other cancer therapies and discuss possible clinical implications.

## Simple Summary

Recent scientific evidence has revealed that microbiota composed of mycoplasma, bacteria, fungi, viruses, and other microbes inhabit the human body in its various tissues and organs and that these microorganisms have many roles in human physiology and pathology. The gut microbiota is the most widely studied so far, and its nature has been clearly correlated with a number of various physiological and pathological conditions, as well as responses to various therapies. Even tumors have their own microbiota, which conditions their pathological traits, immunological profile, and response to therapies. Strategies to combat tumors should therefore rationally take into account their microbial composition. Among a vast variety of available tools to manipulate the microbiota in the human body, antibiotic molecules are an obvious possibility. Some of them have recognized direct effects on microbes but also on human cells and cancer cells in particular. This paper aims to review relevant preclinical and clinical data about the antitumor properties of common antibiotics (excluding specific anticancer antibiotics) and their interactions with current standard anticancer therapies, in order to provide researchers with a tool for designing novel experiments and clinical trials.

## 1. Introduction

Antibiotics belonging to the class of intercalating agents are already part of the standard treatment of different types of hematological and solid tumors. Their anticancer effect has been recognized in causing single- or double-strand DNA breaks by the release of reactive oxygen species (ROS) and by trapping topoisomerase II. The most utilized drugs in this class are anthracyclines in breast cancers and sarcomas, bleomycin in testis cancer, and mitomycin C and D in anal squamous cell carcinomas and other cancers [1].

Antibiotic treatment is known for modulating gut microbiota and augmenting the efficacy of chemotherapy in various types of cancers, especially those of the gastrointestinal tract. In retrospective studies conducted by Imai et al., patients with colorectal and gastric cancer who were treated with different types of antibiotics for non-oncological reasons and received chemotherapy with oxaliplatin, another conventional drug for standard treatments of gastrointestinal cancers, had favorable results in terms of response rate and progression-free survival [2,3]. Modulation of gut microbiota with antibiotics could also reduce the neuropathic pain caused by chemotherapy, a debilitating neurological symptom that is difficult to control even with conventional analgesic drugs, demonstrating that gut microbiota could be involved in the pathogenesis of neuropathic pain [4].

Apart from the role of the gut microbiota, recent research has revealed that tumors are not sterile environments. Various studies have documented the presence of bacteria, fungi, and viruses within different tumor types [5,6,7,8,9,10,11]. The composition of intratumoral microbiota varies significantly between tumor types and even among patients with the same type of cancer. Tumor microbiota vary also with the tumor stage, indicating that different microbial populations may play specific roles in cancer natural history [12,13]. This diversity suggests that intratumoral microbiota may be influenced by multiple factors, including the tumor microenvironment (TME), the host immune response, and prior treatments.

Intratumoral microbiota may even contribute to cancer resistance by producing immunosuppressive molecules or recruiting regulatory immune cells, thereby helping the tumor evade immune surveillance; by metabolizing chemotherapeutic drugs, thereby reducing their efficacy; and by creating biofilms within tumors that can act as physical barriers that prevent the penetration of chemotherapeutic agents [14,15,16].

The interaction between intratumoral microbiota and cancer cells is complex and multifaceted. Microbes within tumors can influence cancer progression through several mechanisms: (1) host immune response modulation, either enhancing antitumor immunity or promoting immune evasion by the tumor; (2) alteration of nutrient availability and the metabolic environment within the tumor, potentially affecting cancer cell growth and survival; (3) direct interaction between bacteria and cancer cells, influencing cellular behaviors such as proliferation, apoptosis, and invasion [17,18,19]. As an example, *Fusobacterium nucleatum*, an anaerobic Gram-negative oral commensal bacterium, is known for having a key role in promoting tumorigenesis of several tumor types, particularly in colorectal cancer (CRC) and pancreatic ductal adenocarcinoma (PDAC), by modulating specific signaling pathways [20,21,22,23,24]. There is also evidence that the same bacterium is one of the components of the TME most responsible for promoting metastasis and in developing acquired resistance to chemotherapy and immunotherapy in CRC [25,26,27].

By altering intratumoral microbiota, antibiotics can make the tumor more susceptible to chemotherapy, highlighting a promising combinatorial approach that may lead to a more effective reduction of tumor burden and an increased survival rate. In one notable example, Gammaproteobacteria (*Escherichia coli*) have been shown to metabolize gemcitabine, a common chemotherapeutic drug utilized in the treatment of cholangiocarcinoma, bladder, and pancreatic cancer, leading to drug resistance. For this reason, many preclinical studies are focusing on the potential combinatorial approaches to increase gemcitabine’s anticancer effect [28,29,30]. Another well-known combinatorial strategy has been explored in order to improve the efficacy of chemotherapy combined with metronidazole [31]. Metronidazole has been used in combination with other therapies to disrupt intratumoral microbiota and improve therapeutic outcomes. Metronidazole targeting *Fusobacterium nucleatum*, the principal component of CRC microbiota, is able to suppress tumor relapse and liver metastasis by regulating gut microbiota in experimental mice [32]. With this rationale, a prospective double-blind randomized clinical trial is now ongoing with the aim of confirming the positive effect of long-term administration of metronidazole in terms of the reduction of the incidence of liver metastasis following surgery in patients with CRC [33].

Interestingly, even common anticancer drugs could have effects on various microbiota. For instance, the fluoropyrimidine drug 5-fluorouracil (5-FU), a very common chemotherapeutic drug utilized in gastrointestinal and head and neck cancers, has an antimicrobial effect against *Fusobacterium nucleatum* [34].

**Figure 1:**
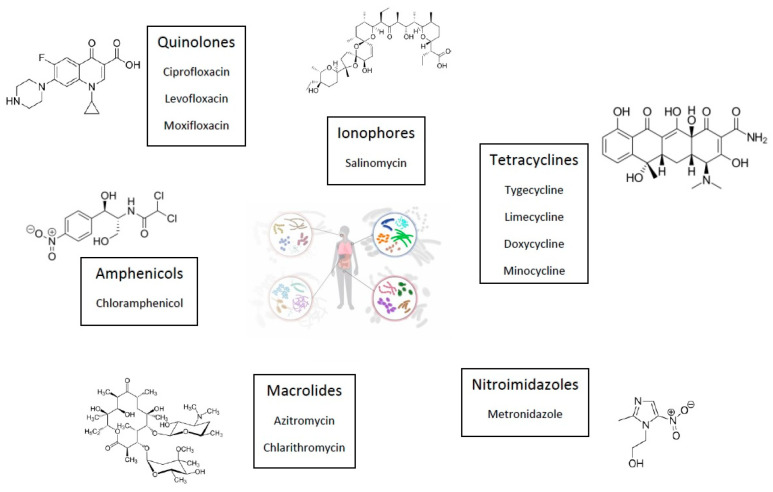
Main common antibiotic classes and specific molecules involved in this study. Their bioavailability in various body districts and complex interaction with different compositions of local microbiota make it very difficult to predict their antitumor and systemic effects. Preclinical and clinical evidence has therefore been empirically reviewed.

Common antibiotics (Figure 1) not only act upon tumor-associated microbes but also have specific effects on cancer cells. Doxycycline and salynomicin activity, for instance, is related to their modulation of the mitochondria with the increased generation of reactive oxygen species (ROS) in fast-cycling cancer stem cells [35].

In this review, we have searched for preclinical studies that have analyzed and tested the antimicrobial and anticancer activity of various common and/or new antibiotics and how they can be applied with cancer therapies (Table 1).

We have collected and reviewed studies highlighting the potential synergistic effects of antibiotics and current cancer therapies. We are also aware that treatment with antibiotics could be the cause of various adverse effects, such as intestinal dysbiosis with diarrhea, due to the inhibition of advantageous bacterial groups, such as *Lactobacillus* and *Bifidobacterium,* and therefore, their application in clinical practice may be problematic and may require treatment or prophylaxis, for example, by combining probiotics and prebiotics [1,36].

## 2. Synergy of Antibiotics with Chemotherapy

Many preclinical studies have reported a synergistic effect between antibiotics and chemotherapeutic drugs in different types of cancers [37,38,39,40,41,42,43,44].

Quinolones

**Figure 2:**
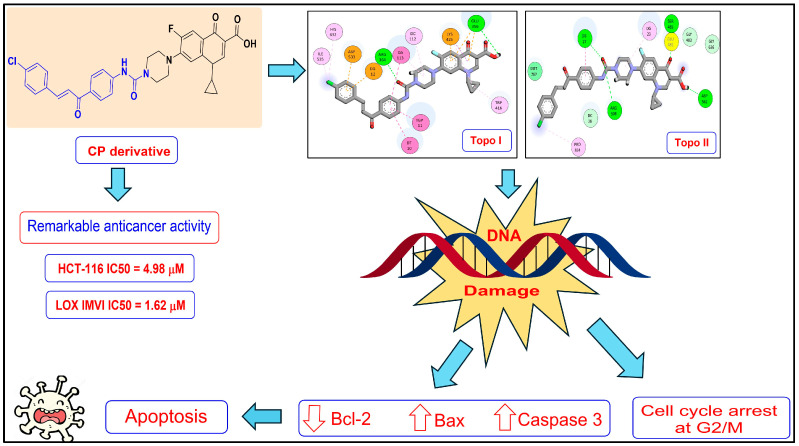
Ciprofloxacin novel derivatives with remarkable anticancer activity are mediated by high-affinity interaction with Topoisomerase enzymes. Reproduced with permission from https://www.mdpi.com/1420-3049/29/22/5382 [52]. Accessed on 20 December 2024.

Fluoroquinolones, a common class of broad-spectrum antibiotics, which inhibit topoisomerases (Figure 2), have been investigated for their potential to disrupt microbial biofilms within tumors, thereby enhancing the penetration and efficacy of chemotherapeutic agents.

As they are the most prescribed antibiotics in genitourinary (GU) infections, ciprofloxacin and levofloxacin have also been studied in bladder and prostate cancer cell lines. Both of them have exhibited toxic effects on tested cell lines and shown an increase in late apoptotic cells and an inhibition of the cell cycle, mainly in the S phase [45]. In addition to the possibility of using them in supportive therapy, quinolones may also have an important role in preventing relapses, especially for bladder cancer [46].

Ciprofloxacin has also been employed in many preclinical studies to confirm that quinolones have a synergistic effect with chemotherapeutic drugs in GU cancers [37,38,39]. Another preclinical study has evaluated ciprofloxacin for its ability to reverse multidrug resistance (MDR) caused by the overexpression of ABCB1, one of the major drug efflux transporters. Ciprofloxacin significantly potentiated the cytotoxic effects of ABCB1 substrates in ABCB1-overexpressing cells. Furthermore, ciprofloxacin increased the intracellular accumulation and decreased the efflux of [^3^H]-paclitaxel without altering the expression of ABCB1 [40]. Ciprofloxacin has also been used in preclinical studies with melanoma [47], breast cancer [48], and pancreatic cancer (together with moxifloxacin) [41], demonstrating that it can trigger apoptosis through the activation of different signaling pathways, depending on the tumor type, and it can sensitize tumor cells to chemotherapy.

A recent preclinical study showed that ciprofloxacin inhibited liver tumor cell growth and proliferation by promoting macrophage polarization toward the M1 inflammatory type. This was seen by monitoring the expression of CD86 (inflammation marker on the macrophage surface) and the production of inflammatory cytokines (TNFα and IL-1β), both augmented during the treatment with ciprofloxacin [49]. This is one of the few studies that demonstrates and underlines the importance of the anticancer effect of an antibiotic not only on tumor cells but also on the tumor microenvironment.

A phase II randomized clinical trial is now ongoing with the aim of confirming the antitumor effect, and a survival benefit, with the addition of levofloxacin to the standard first-line treatment of gemcitabine and nanoparticle-albumin-binding (nab)-paclitaxel for stage IV pancreatic adenocarcinoma [2].

Dual-function lipophilic fluoroquinolones [50] and new triazole ciprofloxacin hybrid compounds with an antimicrobial, antiproliferative, and antioxidant function have been tested to exploit the anticancer properties of quinolones. In a preclinical study, Alaaeldin et al. developed a chemically derived ciprofloxacin chalcone and found it significantly inhibited proliferation, colony formation, and cellular migration; promoted the expression of apoptotic proteins (p53, PUMA, NOXA); and downregulated the epithelial–mesenchymal transition (EMT) against MCF-7 and MDA-MB-231 breast cancer cell lines [51]. These studies point to this class of antibiotics having the potential to create “smart drugs” that are as cytotoxic as conventional chemotherapeutic drugs, but with fewer side effects, such as ciprofloxacin derivatives with increased anti-Topoisomerase activity.

Tetracyclines

In recent years, research on tetracycline antibiotics has gradually elucidated their anticancer effects. Evidence indicates that they have anticancer properties and are able to impact cancer progression through different mechanisms, such as antiproliferation, anti-metastasis, and the promotion of autophagy or apoptosis.

As an example, tigecycline has been used against gastric cancer and multiple myeloma preclinical models and induced cellular autophagy through the conversion of microtubule-associated protein light chain 3-I (LC3I) to LC3II and the fragmentation of autophagic substrate sequestrosome-1 (SQSTM1)/p62 [50,53]. In another study, Jia et al. demonstrated that tigecycline could cause a reduction of B-cell lymphoma-2 (Bcl-2) levels in NSCLC cancer cell lines [54]. Tigecycline has also been studied in pancreatic adenocarcinoma [55] and in melanoma [54], which has inhibited EMT through the downregulation of E2 cyclin and the p21 CIP1/Waf1 complex, respectively. Several preclinical studies have also demonstrated the capacity of tigecycline to interfere with cellular metabolism, induce mitochondrial dysfunction, and inhibit mitochondrial translation [56,57,58].

Another example is doxycycline, which has been reported to modulate the tumor microenvironment, support the activation of an antitumor immune response, and reduce drug resistance and protumorigenic bacteria populations. Many preclinical studies have shown its potential to induce cellular apoptosis [59], impair mitochondrial function [60,61,62], and inhibit migration and invasion by reversing the EMT process and downregulating matrix metallo-proteinases secretion (MMP-2, MMP-9) [63]. Doxycycline can also affect the integrins/focal adhesion kinase (FAK) pathway, which is a key mechanism for developing metastasis in breast cancer, pancreatic cancer, and melanoma [64,65,66]. Phosphatidylinositol 3-kinase (PI3K)/Akt and Wnt/β-catenin are two other signaling pathways involved in processes of cellular growth, proliferation, and metastasis inhibited both by tigecycline and doxycycline [67,68,69,70,71,72,73,74].

**Figure 3:**
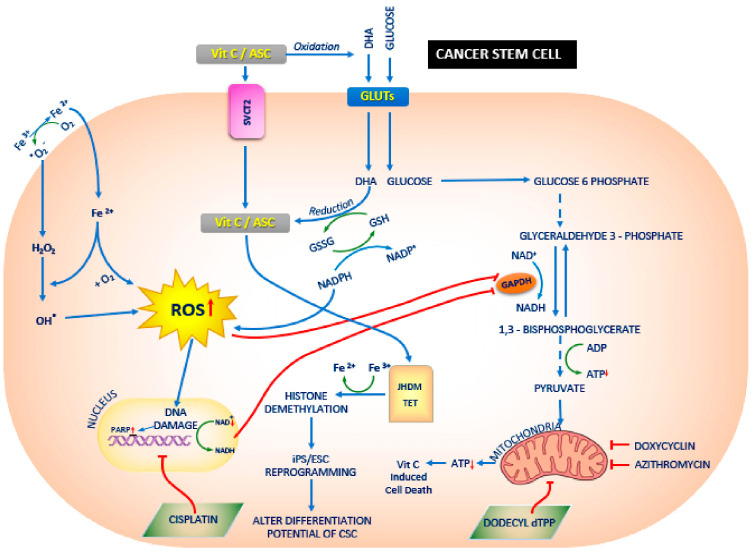
Doxycyclin and azithromycin inhibition of mitochondrial activity leads to increased ROS production, inducing DNA damage and apoptosis in cancer stem cells. These effects can be further potentiated by various substances, such as vitamin C. Reproduced with permission from https://www.mdpi.com/2218-273X/10/1/79 [75]. Accessed on 20 December 2024.

By inhibiting mitochondrial activity and also increasing reactive oxygen species generation, doxycycline and azithromycin strongly can synergize with other agents against cancer stem cells. This mechanism may be further potentiated by ascorbate (vitamin C) (Figure 3).

Minocycline may inhibit the NF-kB pathway, as demonstrated in ovarian cancer preclinical models [76,77].

It is important to highlight that, for these three tetracycline antibiotics, there is evidence that they can also enhance sensitivity to chemotherapeutic drugs in various types of cancer [42,43,44,78].

Moreover, in a preclinical study conducted by Akhunzianov et al. using a breast cancer MCF-7 cell line model, it was demonstrated that the application of five antibiotics (tetracycline, doxycycline, azithromycin, erythromycin, and chloramphenicol) could effectively inhibit breast cancer cell growth, but their killing mechanism was defective in hypoxic conditions, underlining that this condition is an obstacle not only for anticancer immune response but also for antibiotics [79].

Macrolides

Macrolides, a group of broad-spectrum widely used antibiotics, have been studied for their property of inhibiting autophagy and mitophagy, self-digestive processes that maintain cellular homeostasis by recycling intracellular components. While in normal cells autophagy plays a role of scavenger by removing damaged mitochondria that produce ROS, and thus it is important to prevent tumorigenesis, in cancer cells, it seems to have an adjuvant role in terms of adaptation to hostile environment conditions, like hypoxia. Therefore, blocking autophagy has become a new strategy for impairing the metabolism of cancer cells at a critical point, and this strategy can be realized thanks to macrolides (azithromycin being the most utilized). This has been demonstrated in various types of solid tumors, like head and neck, lung, and pancreatic cancer cell lines [80,81,82,83].

Clarithromycin has been tested with cisplatin, a common drug applied in various types of cancer, in vitro and in vivo against ovarian cancer cell lines; it has been found that clarithromycin reduces tumor growth by limiting endogenous antioxidant enzyme expression and increasing the levels of ROS, thereby potentiating the cytotoxic effect of cisplatin [84].

Chloramphenicol

Chloramphenicol, a Gram-negative-targeting antibiotic, is able to induce autophagy; in a preclinical study conducted by Hsu et al., chloramphenicol decreased the levels of hypoxia inducible factor 1 (HIF-1α), vascular endothelial growth factor (VEGF), and glucose transporter 1 (GT-1) and induced autophagy in non-small cell lung cancer cell lines [85]. Even in glioblastoma patient-derived stem-like cells, chloramphenicol was able to induce ferroptosis, which could be the underlying mechanism through which it acts [86].

Salinomycin

**Figure 4:**
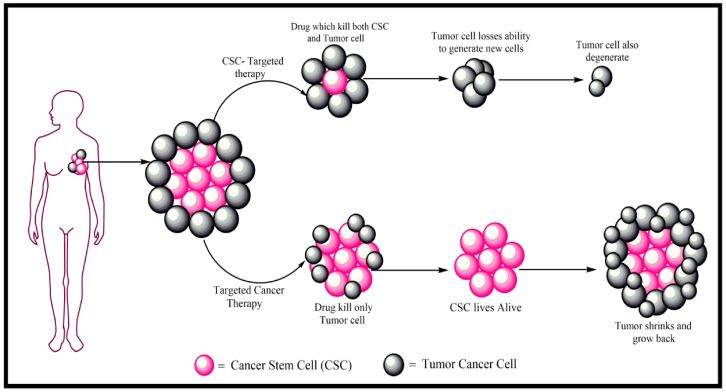
Schematic representing a chemotherapeutic drug approach to targeting cancer stem cells (CSCs) as a potential treatment for cancer. Salinomycin has shown specific preclinical activity against CSCs. Reproduced with permission from https://www.mdpi.com/2673-8937/3/2/16 [91]. Accessed on 20 December 2024.

The ionophore and coccidiostat salinomycin, developed to target Gram-positive bacteria (in particular, methicillin-resistant *Staphylococcus aureus* and *Epidermidis*), has shown anticancer activity, attributed to lysosomal iron sequestration and permeabilization and the mechanism of ferroptosis, due to the release of ROS [35,87,88]. Moreover, it has been shown to inhibit angiogenesis and reduce breast cancer growth in vitro and in vivo [89] and to modulate epigenetic mechanisms in colon cancer cell lines [90] (Figure 4).

## 3. Synergy of Antibiotics with TKIs

**Figure 5:**
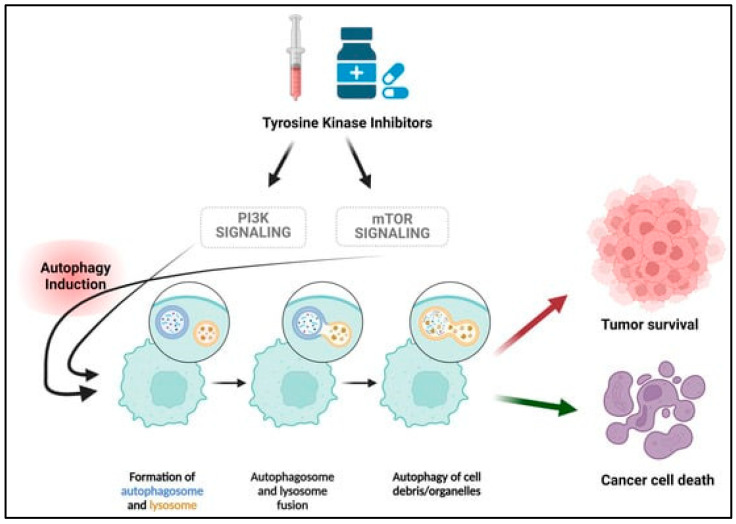
Autophagy induced by Tetracyclines and Macrolides synergize with Tyrosine Kinase Inhibition, tilting the balance toward cancer cell death instead of cancer cell resistance to antiproliferative agents. Reproduced with permission from https://www.mdpi.com/2072-6694/16/17/2989 [98]. Accessed on 20 December 2024.

A synergistic effect between antibiotics and tyrosine-kinase inhibitors (TKIs) has also been described (Figure 5). Given that many TKIs induce autophagy in cancer cells, regardless of their original target, and considering the potential cytoprotective role of autophagy for cancer cells, blocking TKI-induced and proteasome inhibitors-induced autophagy with macrolides may enhance cytotoxicity via non-apoptotic cell death [81,92,93,94,95]. Another preclinical study conducted by Chen et al. on lung cancer cell lines resistant to EGFR-TKIs demonstrated that lymecycline, a semisynthetic derivative of tetracycline, was able to reverse cancer cells’ resistance to icotinib, an EGFR-targeting TKI, by inhibiting EGFR phosphorylation and growth factor receptor-bound protein 2 (GRB2)-mediated AKT/ERK/STAT3 signaling pathways, resulting in a synergistic effect with these two drugs [95].

Two novel targets have been identified by the group of Kositza et al. on bladder cancer cell lines T24, RT112, and UMUC3: MCM6 and KIFC1 are two of the targetable genes that confer resistance to CDK4/6 inhibitors, and the combination of palbociclib and other MCM6/KIFC1 inhibitors, including ciprofloxacin, resulted in a synergistic effect in terms of delayed tumor growth [96].

Also, the blood–brain barrier penetration of sunitinib has been shown to be improved by co-administration with ciprofloxacin [97].

## 4. Synergy of Antibiotics with Immunotherapy

Despite some preclinical evidence, there is little evidence about the synergistic effect between antibiotics and current immunotherapies in clinical use, in particular, monoclonal antibody PD1/PDL1 immune checkpoint inhibitors.

A synergistic effect between antibiotics and adoptive cell therapy has not been described, but in this field, antibiotics potentially could be applied as modulators; as an example, doxycycline was tested in a preclinical study with CD147-targeting chimeric antigen receptor T lymphocyte (CAR-T) cells against hepatocarcinoma, and it effectively controlled CAR-T cells’ toxicity and facilitated effector activity [99].

As ionic concentrations in the tumor microenvironment could strongly impair the effector function of T cells, modulating it by the ionophore function of macrolide antibiotics could help unleash the activity of T cells in the tumor microenvironment [100].

Many preclinical studies have demonstrated a potentiated immune response against cancer with the administration of certain antibiotics. For example, doxycycline increases the MHC-I expression on the tumor cell surface (one of the mechanisms of tumor cell immune evasion) by inhibiting autophagy, thereby reactivating the immune system and providing a rationale to explore combinatorial therapy with immunotherapeutic drugs [101]. Moreover, the interaction between various tetracyclines antibiotics and peripheral T cells from healthy donors enhanced T-cell cytotoxicity through granzyme B production and CD8+ T-cells proliferation, ameliorating their infiltration in lung cancer tumor tissues, indicating another potential for antibiotics’ combination with immunotherapy [102].

On the other side, antibiotic-induced gut microbiota dysbiosis affects the efficacy of various cancer treatments, in particular, immunotherapy. Some studies have documented a detrimental effect of commonly used antibiotics on immunotherapies, such as Immune Checkpoint Inhibitors (ICIs) and CAR-T cells [103,104]. Considering the frequent infectious diseases and the consequent abuse of common antibiotics, this is a considerable issue for patients affected by tumors that could be treated with immunotherapies, and adding other antibiotics in synergy with immunotherapeutic drugs could potentially worsen the outcome for these patients. For this reason, further preclinical studies are needed to clarify whether the drawbacks outweigh the advantages concerning this combination.

## 5. Innovative Antibiotic Strategies

Given that the majority of cancerogenic bacteria are intracellular, to enhance the intracytoplasmic delivery of metronidazole into PDAC cells infected by *Fusobacterium nucleatum*, Duncan et al. combined metronidazole treatment with electro-antibacterial therapy (EAT), an electroporation technique based on electric pulses that increases the permeability of infected cells [105]. This combinatorial technique led to the elimination of 99% of bacteria composition and could represent a new paradigm for pancreatic cancer therapy. The rationale of this study is based on previous works that investigated the possibility to facilitate antibiotic action and bacterial inactivation [106,107].

**Figure 6:**
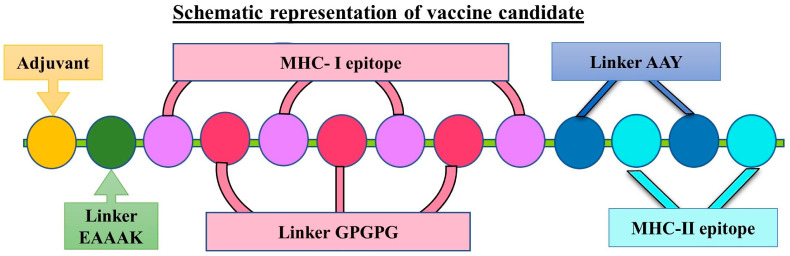
Schematic representation of the multi-epitope-based vaccine designed by linking the MHC-I and MHC-II epitopes of *F. nucleatum* with the different linkers and adjuvant. Reproduced with permission from https://www.mdpi.com/2076-393X/11/3/525 [108]. Accessed on 20 December 2024.

Investigating the combination of antibacterial molecules against *Fusobacterium nucleatum,* with the increased knowledge of the molecular mechanisms involved in oncogenesis, Padma et al. identified the fibroblast activation protein-2 (Fap2) of *Fusobacterium nucleatum* as an important antigen for developing a colorectal cancer vaccine [108] (Figure 6). Also, the polysaccharide D-galactose-β(1-3)-N-acetyl-D-galactosamine (Gal-GalNAc) from CRC tissues has proven to be a promising antigen targetable with a dendritic cell (DC)-based vaccine against CRC cells, adjuvanted by the action of tubeimuside I, a saponin substance that inhibited intracellular *Fusobacterium nucleatum*’s infection [109].

Nanotechnology is an active and promising field where researchers are designing antibiotic-based nanoplatform structures with the aim of increasing the chemosensitivity and the cytotoxicity of cancer cells or to potentiate the immune response [101,110,111,112]. Antibiotic nanoparticles have been developed with the aim of enhancing infiltration inside tumor masses in order to kill bacteria inside tumor cells. Gao et al. combined metronidazole and fluorouridine with nanoparticles to obtain a synergistic effect with a dual-targeting action on both cancer cells and intratumoral microbiota, showing encouraging results in terms of infiltration capacity and balance maintenance of the patient’s microbiota [31].

Size-tunable nanogels for metronidazole targeting *Fusobacterium nucleatum* have also been proposed as an innovative strategy to potentiate antibiotic action [113]. In an innovative preclinical study on *Fusobacterium nucleatum*-infected CRC, model zinc-imidazolate frameworks with doxorubicin loading and folate grafting mixed with metronidazole and encapsulated in size-tunable nanogels were applied. This particular technique permitted a prolonged retention of this product inside the infected tumor cells and promoted a dual-responsive cascade drug release with impressive results [113]. Nanomedicine’s techniques on various types of cancer are also being applied to salinomycin [114,115].

Metal complexes with antioxidant and antimicrobial properties have long been used to treat skin infections in medicine. Silver complexes with antimicrobial and antifungal agents have shown interesting results in vitro against various types of cancer, not only because of their high selectivity and cytotoxicity against bacterial and tumor cells, but also for their safety and biocompatibility [116,117,118,119,120,121,122]. Strategies for antibacterial-enhanced chemotherapy and immunotherapy are currently an area of active research [110,112,123].

The activity of certain antibiotics upon mitochondria, which has been researched by the group of Lisanti and others as essential for cancer cell stemness, could be the subject of important future research. Acting upon microorganisms and mitochondria, antifungals such as miconazole or itraconazole, as well as other substances, could act as metabolic modulators [124] and could impair the metastatic process, reduce malignant mutations with a poor prognosis, and inhibit the immortal phenotype of cancer cells [62,112,125,126,127].

## 6. Clinical Trials

Currently, there are multiple ongoing clinical trials utilizing various antibiotics, including clarithromycin, doxycycline, metronidazole, ciprofloxacin, azithromycin, levofloxacin, and tigecycline. Table 2 shows an array of active clinical trials recorded in the ReDO_Trials database [128], exploring the repurposing of antibiotics for oncology. Antibiotics like metronidazole and ciprofloxacin are being evaluated for their ability to alter gut microbiota to improve immunotherapy or chemotherapy outcomes. Macrolides like clarithromycin and azithromycin are explored for their anti-inflammatory and immune-modulating roles, particularly in hematological malignancies. Doxycycline is highlighted for its potential to target cancer stem cells, particularly in breast cancer. Many trials combine antibiotics with chemotherapeutic agents (e.g., gemcitabine, nab-paclitaxel) or novel treatments (e.g., CAR T-cell therapy), suggesting synergy between antibiotics and standard or emerging treatments. If proven effective, antibiotic repurposing could offer cost-effective, widely accessible adjunctive therapies for cancer, leveraging decades of safety data for these drugs. However, there is a need for caution to avoid long-term antimicrobial resistance and other unintended consequences.

## 7. Conclusions and Perspectives

Antibiotics used to combat microbial infections in the general population, even in cancer patients, have various effects in addition to those for which they are prescribed. Antibiotic molecules can act upon microorganisms inside various tissues and organs in the human body simultaneously, including in the tumor microenvironment. Targets are also present in the gut microbiota, a newly recognized fundamental element in modern oncology and immunology. Most of these interactions remain currently unexplored, and scientific research in this area is therefore strongly warranted, both to better evaluate the safe use of these drugs in cancer patients and to exploit their synergism with anticancer therapies.

Much of the available preclinical knowledge on the anticancer potential of antibiotics, however, has not been translated into clinical trials yet. In particular, novel formulations specifically developed to target tumor-associated microbes have great potential to be translated into effective and safer anticancer therapies in the near future

In this paper, we reviewed the known evidence of synergistic effects of widely available antibiotics with various chemotherapeutics, tyrosine kinase inhibitors, and immunotherapeutics. The literature shows that such drugs can indeed display synergy with various anticancer therapies. In preclinical studies, some mechanisms have begun to be elucidated, and clinical trials are currently ongoing. It is hoped that the results of these trials will reveal whether cancer patients can achieve some benefit from adding inexpensive and manageable antibiotics to their anticancer therapies. Concerns regarding possible increased toxicities or adverse effects on the efficacy of some immune checkpoint inhibitors must also be addressed in order to properly assess the risks and benefits of incorporating concomitant antibiotics into standard-of-care anticancer regimens. However, the evidence summarized here suggests that further research, including clinical studies, is warranted.

## Figures and Tables

**Table 1:** Synergy of common antibiotics with anticancer treatments.

	Chemotherapy	Targeted Therapy	Immunotherapy
Quinolones	Reverse MDR by altering ABCB1 expression, induce apoptosis	Delay tumor’s growth by MCM6/KIFC1 inhibition in combination with Palbociclib and improve sunitinib penetration through the blood–brain barrier	/
Tetracyclines	Inhibit phosforilation signaling pathways; induce autophagy/apoptosis and mitochondrial damage; inhibit EMT, MMPs, and NF-kB pathway	Inhibit EGFR phosphorylation and GRB2 signaling pathway	Potentiate immune response and CAR-T cells activity by inhibiting autophagy
Macrolides	Induce autophagy and increase platinum sensitivity by limiting endogenous antioxidant enzyme expression and increasing the levels of ROS	Block TKIs-induced autophagy	/
Metronidazole	Targets *Fusobacterium nucleatum* and regulates gut microbiota	/	/
Chloramphenicol	Induces autophagy and decreases the levels of HIF-1α, VEGF, and GT-1	/	/
Salinomycin	Induces ferroptosis, inhibits angiogenesis, and modulates epigenetic mechanisms	/	/
Cephalosporines	Promote extrinsic apoptotic signaling	/	/

**Table 2:** Ongoing clinical trials testing antibiotics as part of anticancer treatment.

Clinical Trial Number	Title	Conditions	Country	Drug	PhaseCode
ChiCTR2000029245	The efficacy and safety of the combination of transcatheter arterial chemoembolization with metronidazole in hepatocellular carcinoma	Hepatocellular Carcinoma	China	Metronidazole	Other
ChiCTR1800014946	Thalidomide, clarithromycin, and dexamethasone regimen for patients with newly diagnosed multiple myeloma	Multiple Myeloma	China	Clarithromycin	Other
ChiCTR-IOR-17010695	The significance of minimal residual disease examination on the multiple myeloma maintain treatment	Multiple Myeloma	China	Clarithromycin	Other
ACTRN12620000815965	Phase II trial of doxycycline with radiotherapy for rectal cancer	Rectal Cancer; Cancer–Bowel–Back passage (rectum) or large bowel (colon)	New Zealand	Doxycycline	Phase 2
NCT05462496	Modulation of the gut microbiome with pembrolizumab following chemotherapy in resectable pancreatic cancer	Pancreatic Cancer	United States	Ciprofloxacin Metronidazole	Phase 2
NCT04523987	A pilot study of ciprofloxacin plus gemcitabine and nab-paclitaxel chemotherapy in patients with metastatic pancreatic ductal adenocarcinoma	Metastatic Pancreatic Ductal Adenocarcinoma	Singapore	Ciprofloxacin	Phase 1
NCT02387203	Antibiotic treatment and long-term outcomes of patients with pseudomyxoma peritonei of appendiceal origin	Pseudomyxoma Peritonei|Appendiceal Neoplasms	United States	Clarithromycin	Phase 2
NCT01745588	Autologous stem cell transplant with pomalidomide (cc-4047^®^) maintenance versus continuous clarithromycin/pomalidomide/dexamethasone salvage therapy in relapsed or refractory multiple myeloma	Multiple Myeloma	United States	Clarithromycin	Phase 2
NCT04302324	A phase II study of daratumumab, clarithromycin, pomalidomide, and dexamethasone (d-clapd) in multiple myeloma patients previously exposed to daratumumab	Multiple Myeloma|Refractory Multiple Myeloma|Relapse Multiple Myeloma	United States	Clarithromycin	Phase 2
NCT02542657	Ixazomib with pomalidomide, clarithromycin, and dexamethasone in treating patients with multiple myeloma	Myeloma	United States	Clarithromycin	Phase 1/2
NCT04287660	Study of BiRd regimen combined with BCMA CAR T-cell therapy in newly diagnosed multiple myeloma (MM) patients	Multiple Myeloma	China	Clarithromycin	Phase 3
NCT01559935	Carfilzomib, clarithromycin (Biaxin^®^), lenalidomide (Revlimid^®^), and Dexamethasone (Decadron^®^) [Car-BiRD] therapy for subjects with multiple myeloma	multiple myeloma	United States	Clarithromycin	Phase 2
NCT02343042	Selinexor and backbone treatments of multiple myeloma patients	Multiple Myeloma	United States	Clarithromycin	Phase 1/2
NCT03031483	Clarithromycin + Lenalidomide Combination: a full oral treatment for patients with relapsed/refractory extranodal marginal zone lymphoma	Mucosa Associated Lymphoid Tissue (MALT) Lymphoma	Italy	Clarithromycin	Phase 2
NCT02875223	A safety and efficacy study of CC-90011 in participants with relapsed and/or refractory solid tumors and non-Hodgkin lymphomas	Lymphoma, Non-Hodgkin|Neoplasms	France	Itraconazole Rifampicin	Phase 1
NCT03076281	Metformin hydrochloride and doxycycline in treating patients with head and neck squamous cell carcinoma that can be removed by surgery	Larynx|LIP|Oral Cavity|Pharynx	United States	Doxycycline	Phase 2
NCT01820910	Phase II trial of first-line doxycycline for ocular adnexal marginal zone lymphoma treatment	Marginal Zone Lymphoma of Ocular Adnexal	United States	Doxycycline	Phase 2
NCT03116659	CTCL directed therapy	Lymphoma, T Cell, Cutaneous	United States	Doxycycline	Phase 1
NCT04264676	Study of oral metronidazole on postoperative chemotherapy in colorectal cancer	Colorectal Cancer Stage II|Colorectal Cancer Stage III	China	Metronidazole	Phase 1
NCT05720559	Early blocking strategy for metachronous liver metastasis of colorectal cancer based on pre-hepatic CTC detection	Preventive Effect of Quintuple Therapy on Metachronous Liver Metastases in Patients With Colorectal Cancer	China	Metronidazole	Phase 2
NCT05774964	Quintuple method for treatment of multiple refractory colorectal liver metastases	For Patients With Colorectal Cancer Liver Metastases Who Were Not Able to Obtain Curative Surgical Resection. Focused on the Treatment Effect with the Quintuple Method.	China	Metronidazole	Phase 2
NCT06126731	Combination study of antibiotics with enzalutamide (PROMIZE)	Metastatic Castration-Resistant Prostate Cancer (mCRPC)	United Kingdom	Ciprofloxacin Metronidazole	Phase 1/2
2020-003152-33	A phase II trial of long-term intravenous treatment with bi-weekly Azithromycin in patients with gastric lymphoma of the mucosa-associated lymphoid tissue (MALT-lymphoma)	Gastric MALT Lymphoma	Austria	Azithromycin	Phase 2
2019-004074-25	A phase II open-label, randomized, controlled, pre-surgical feasibility study of antibiotic combinations in early breast cancer	We investigated, in a population of patients with breast cancer, the combined effect of azithrocyn, docyciclin, and vitamin C on biomarkers associated with cell proliferation	Italy	Azithromycin	Phase 2
2016-000871-26	A phase II open-label, randomized, controlled, pre-surgical feasibility study of antibiotic combinations in early breast cancer	Breast cancer—Stages 1–2 to or Stage 3 that is candidate for primary surgery	Italy	Doxycycline	Phase 2
ChiCTR2100047608	Clarithromycin added to pomadomide-cyclophosphamide-dexamethasone (Cla-PCd) versus pomadomide cyclophosphamide-dexamethasone (PCd) in relapsed or refractory multiple myeloma: a prospective, multicenter, randomized, controlled clinical trial	Multiple Myeloma	China	Clarithromycin	Phase 4
ChiCTR2100046201	A prospective, multicenter, randomized, controlled study on whether long-term oral antibiotics can effectively reduce the incidence of postoperative tumor recurrence and metastasis in patients with colorectal cancer	Colorectal Cancer	China	Metronidazole	Not Available/Missing
RPCEC00000367	Doxycycline for prostate cancer	Prostate Cancer; Prostatic Neoplasms; Genital Neoplasms, Male; Urogenital Neoplasms; Prostatic Diseases; Genital Diseases, Male; Male Urogenital Diseases	Mexico	Doxycycline	Phase 2
ChiCTR2100054650	A phase II clinical study evaluating the efficacy and safety of Fruquintinib combined with azithromycin liposome in patients with Platinum resistant ovarian cancer	Ovarian Cancer	China	Azithromycin	Phase 4
JPRN-jRCTs021230005	A randomized phase 2 study assessing the efficacy and safety of levofloxacin added to the GEM/nabPTX combination therapy in patients with advanced pancreatic cancer(T-CORE2201)	Pancreatic Cancer	Japan	Levofloxacin	Phase 2
ACTRN12623001164684	Assessing treatment effectiveness of the ‘Repurposing-Drugs-in-Oncology’ (ReDO) protocol for cancer: The ReDO cancer treatment study	Cancer-Any cancer	Australia	Doxycycline	Other
NCT02874430	Metformin Hydrochloride and Doxycycline in Treating Patients With Localized Breast or Uterine Cancer	Breast Carcinoma, Endometrial Clear Cell Adenocarcinoma, Endometrial Serous Adenocarcinoma, Uterine Corpus Cancer, Uterine Corpus Carcinosarcoma	United States	Doxycycline	Phase 2
NCT04063189	Clinical Trial of Clarithromycin, Lenalidomide and Dexamethasone in the Treatment of the First Relapsed Multiple Myeloma	Multiple Myeloma in Relapse	China	Clarithromycin	Phase 2
NCT02575144	GEM-CLARIDEX: Ld vs. BiRd	Multiple Myeloma	Spain	Clarithromycin	Phase 3
NCT03962920	Personalized Treatment of Urogenital Cancers Depends on the Microbiome	Microbial Disease	Denmark	Tigecycline	Other
NCT06452394	NEODOXy: Targeting Breast Cancer Stem Cells With Doxycycline	Breast Cancer	Switzerland	Doxycycline	Phase 2

## Data Availability

No new data created.
